# Supplementary material for: Regional Variations in Outpatient Antibiotic Prescribing in Germany: A Small Area Analysis Based on Claims Data
Source: Antibiotics (Basel). 2022 Jun 22;11(7):836. doi: 10.3390/antibiotics11070836 (PMC9312140; doi:10.3390/antibiotics11070836)
Supplement: Supplementary file 1 [file antibiotics-11-00836-s001.zip › antibiotics-1748917-supplementary.pdf]

SUPPLEMENTARY MATERIALS

Table S1: Classification of antibiotics into subgroups. 2

Table S2a: Study population, antibiotic prescriptions, and standardized prescription rates of antibiotics in 2010 and 2018 among females. 4

Table S2b: Study population, antibiotic prescriptions, and standardized prescription rates of antibiotics in 2010 and 2018 among males. 5

Table S3a: Proportional distribution of antibiotic prescriptions by antibiotic subgroup in 2018 among both sexes. 6

Table S3b: Proportional distribution of antibiotic prescriptions by antibiotic subgroup in 2018 among females. 7

Table S3c: Proportional distribution of antibiotic prescriptions by antibiotic subgroup in 2018 among males. 8

Figure S1: Map of Germany with the 16 federal states. 9

Figure S2: Age-specific prescription rates (prescriptions per 1000 persons/year) of antibiotics by sex in 2018. 10

Figure S3: Age- and sex-standardized prescription rates (prescriptions per 1000 persons/year) of antibiotics among children aged ≤6 years by district in 2018. 11

Figure S4: Age- and sex-standardized prescription rates (prescriptions per 1000 persons/year) of antibiotics among children and adolescents aged 0–17 years by district in 2010. 12

Figure S5: Age- and sex-standardized prescription rates (prescriptions per 1000 persons/year) of antibiotics among children aged ≤6 years by district in 2010. 13

Figure S6: Age- and sex-standardized prescription rates (prescriptions per 1000 persons/year) of antibiotics among adults aged ≥18 years by district in 2010. 14

Figure S7: Age- and sex-standardized prescription prevalence (%) of antibiotics among children and adolescents aged 0–17 years by district in 2018 (see Text S1 below for a description). 15

Figure S8: Age- and sex-standardized prescription prevalence (%) of antibiotics among children aged ≤6 years by district in 2018 (see Text S1 below for a description). 16

Figure S9: Age- and sex-standardized prescription prevalence (%) of antibiotics among adults aged ≥18 years by district in 2018 (see Text S1 below for a description). 17

Text S1: Regional variations in antibiotic prescribing based on the prescription *prevalence* in 2018 (referring to Figures S7–S9). 18

Table S1: Classification of antibiotics into subgroups.

Subgroup		ATC label	ATC code
Combinations of aminopenicillins and antistaphylococcal penicillins	Combinations of aminopenicillins	Ampicillin and beta-lactamase inhibitor	J01CR01
		Amoxicillin and beta-lactamase inhibitor	J01CR02
		Sultamicillin	J01CR04
		Piperacillin and beta-lactamase inhibitor	J01CR05
		Combinations of penicillins	J01CR50
	Other combinations of aminopenicillins and antistaphylococcal penicillins	Beta-lactamase sensitive penicillins, combinations	J01CE30
		Dicloxacillin	J01CF01
		Oxacillin	J01CF04
		Flucloxacillin	J01CF05
Basic penicillins	Amoxicillin	Amoxicillin	J01CA04
	Phenoxymethyl-penicillin	Phenoxymethylpenicillin	J01CE02
	Other basic penicillins	Ampicillin	J01CA01
		Pivmecillinam	J01CA08
		Mezlocillin	J01CA10
		Piperacillin	J01CA12
		Benzylpenicillin	J01CE01
		Propicillin	J01CE03
		Azidocillin	J01CE04
		Benzathine benzylpenicillin	J01CE08
		Benzathine phenoxymethylpenicillin	J01CE10
		Sulbactam	J01CG01
Cephalosporins	First generation	Cefalexin	J01DB01
		Cefazolin	J01DB04
		Cefadroxil	J01DB05
	Second generation	Cefoxitin	J01DC01
		Cefuroxime	J01DC02
		Cefaclor	J01DC04
		Cefotiam	J01DC07
		Loracarbef	J01DC08
	Third generation	Cefotaxime	J01DD01
		Ceftazidime	J01DD02
		Ceftriaxone	J01DD04
		Cefixime	J01DD08
		Cefpodoxime	J01DD13
		Ceftibuten	J01DD14
		Ceftazidime and beta-lactamase inhibitor	J01DD52
	Fourth generation	Cefepime	J01DE01
		Ceftaroline fosamil	J01DI02
Macrolides / lincosamides		Erythromycin	J01FA01
		Spiramycin	J01FA02
		Roxithromycin	J01FA06
		Clarithromycin	J01FA09
		Azithromycin	J01FA10
		Telithromycin	J01FA15
		Clindamycin	J01FF01

Subgroup		ATC label	ATC code
Fluoroquinolones	Group 1 (ciprofloxacin)	Ciprofloxacin	J01MA02
	Group 2 (levo- and moxifloxacin)	Levofloxacin	J01MA12
		Moxifloxacin	J01MA14
	Group 3 (e.g., ofloxacin)	Ofloxacin	J01MA01
		Enoxacin	J01MA04
		Norfloxacin	J01MA06
Metronidazole		Metronidazole	J01XD01
		Metronidazole	P01AB01
Nitrofurantoin / fosfomycin / nitroxoline		Nitrofurantoin	J01XE01
		Nitrofurantoin, combinations	J01XE51
		Fosfomycin	J01XX01
		Nitroxoline	J01XX07
Sulfonamides / trimethoprim		Trimethoprim	J01EA01
		Sulfadiazine	J01EC02
		Sulfamethoxazole and trimethoprim	J01EE01
Tetracyclines		Doxycycline	J01AA02
		Tetracycline	J01AA07
		Minocycline	J01AA08
		Tigecycline	J01AA12
Other		Aztreonam	J01DF01
		Meropenem	J01DH02
		Ertapenem	J01DH03
		Doripenem	J01DH04
		Imipenem and cilastatin	J01DH51
		Streptomycin	J01GA01
		Tobramycin	J01GB01
		Gentamicin	J01GB03
		Neomycin	J01GB05
		Amikacin	J01GB06
		Gentamicin, combinations	J01GB53
		Neomycin, combinations	J01GB55
		Pipemidic acid	J01MB04
		Vancomycin	J01XA01
		Teicoplanin	J01XA02
		Dalbavancin	J01XA04
		Colistin	J01XB01
		Linezolid	J01XX08
		Daptomycin	J01XX09
		All other codes starting with J01 not mentioned	

Table S2a: Study population, antibiotic prescriptions, and standardized prescription rates of antibiotics in 2010 and 2018 among females.

	Age group in years											Overall
	**0–1**	**2–5**	**6–9**	**10–14**	**15–17**	**18–24**	**25–34**	**35–44**	**45–54**	**55–64**	**≥65**	
Study population, n												
2010	66,374	213,030	233,536	342,297	185,823	510,032	877,260	1,031,187	1,282,751	964,732	1,562,203	7,269,225
2018	101,331	307,686	278,922	339,795	215,386	617,433	1,167,824	1,120,831	1,386,353	1,354,565	1,990,225	8,880,351
Antibiotic prescriptions, n												
2010	49,439	221,768	144,129	141,336	123,319	383,794	583,557	648,992	748,055	603,732	1,024,897	4,673,018
2018	36,864	171,886	107,200	86,355	94,062	345,715	588,375	590,507	677,158	722,298	1,140,434	4,560,854
Standardized prescription rates[a]												
2010	593.6	1,042.2	620.6	413.5	671.2	756.3	665.4	639.3	586.1	625.6	665.5	650.8
2018	282.6	558.4	383.1	253.8	436.9	560.7	503.8	526.7	488.4	533.6	576.2	511.0
Change in prescription rate from 2010 to 2018	-52%	-46%	-38%	-39%	-35%	-26%	-24%	-18%	-17%	-15%	-13%	-21%

[a] Prescriptions per 1000 persons/year; age-standardized using the German population on December 31, 2017 as reference.

Table S2b: Study population, antibiotic prescriptions, and standardized prescription rates of antibiotics in 2010 and 2018 among males.

	**Age group in years**											**Overall**
	**0–1**	**2–5**	**6–9**	**10–14**	**15–17**	**18–24**	**25–34**	**35–44**	**45–54**	**55–64**	**≥65**	
Study population, n												
2010	70,185	223,856	247,224	359,160	192,331	503,326	811,404	806,552	1,010,645	770,164	1,122,902	6,117,749
2018	105,982	323,708	295,640	359,127	228,165	661,143	1,156,310	1,023,984	1,123,899	1,090,658	1,438,831	7,807,447
Antibiotic prescriptions, n												
2010	61,618	244,628	140,811	127,770	84,626	210,487	320,968	366,332	428,715	372,394	636,611	2,994,960
2018	45,978	187,274	104,025	79,087	64,810	193,127	331,758	365,863	400,581	439,009	689,411	2,900,923
Standardized prescription rates[a]												
2010	706.2	1,094.6	572.8	356.2	443.1	419.8	396.9	457.1	425.4	483.1	581.9	496.4
2018	343.3	577.9	350.6	220.0	284.4	292.8	286.3	357.8	356.5	403.0	478.6	371.2
Change in prescription rate from 2010 to 2018	-51%	-47%	-39%	-38%	-36%	-30%	-28%	-22%	-16%	-17%	-18%	-25%

[a] Prescriptions per 1000 persons/year; age-standardized using the German population on December 31, 2017 as reference.

Table S3a: Proportional distribution of antibiotic prescriptions by antibiotic subgroup in 2018 among both sexes.

	Age group in years											Overall
	**0–1**	**2–5**	**6–9**	**10–14**	**15–17**	**18–24**	**25–34**	**35–44**	**45–54**	**55–64**	**≥65**	
Standardized prescription rate[a]	*313.6*	*568.5*	*366.5*	*236.3*	*357.8*	*419.9*	*391.4*	*441.5*	*421.9*	*469.2*	*533.8*	*441.9*
Combinations of aminopenicillins	4.6%	3.9%	4.1%	4.6%	5.3%	5.6%	6.1%	6.7%	7.3%	8.0%	9.1%	7.1%
Other combinations of aminopenicillins and antistaphylococcal penicillins	0.0%	0.0%	0.0%	0.1%	0.1%	0.1%	0.3%	0.2%	0.2%	0.1%	0.1%	0.2%
Amoxicillin	38.7%	32.0%	24.1%	22.5%	20.4%	17.7%	17.8%	18.0%	16.4%	15.3%	11.9%	17.0%
Phenoxymethylpenicillin	3.7%	11.3%	16.1%	12.6%	9.4%	7.9%	6.6%	5.4%	3.3%	2.7%	2.1%	5.0%
Other basic penicillins	1.9%	5.4%	6.5%	2.5%	0.3%	0.4%	0.4%	0.4%	0.4%	0.4%	0.6%	0.9%
Cephalosporins, first generation	0.5%	1.3%	2.0%	1.4%	0.6%	0.4%	0.4%	0.3%	0.3%	0.3%	0.3%	0.4%
Cephalosporins, second generation	31.8%	27.8%	25.5%	24.8%	17.6%	14.2%	14.5%	14.7%	13.5%	12.9%	13.0%	15.2%
Cephalosporins, third generation	7.6%	5.3%	4.3%	3.8%	2.8%	2.1%	2.1%	2.2%	2.2%	2.2%	2.6%	2.6%
Cephalosporins, fourth generation	0.0%	0.0%	0.0%	0.0%	0.0%	0.0%	0.0%	0.0%	0.0%	0.0%	0.0%	0.0%
Macrolides / lincosamides	6.9%	9.3%	11.6%	18.5%	22.1%	21.6%	22.2%	23.9%	23.7%	21.7%	15.1%	19.5%
Fluoroquinolones group 1 (ciprofloxacin)	0.1%	0.1%	0.3%	0.9%	3.0%	5.9%	6.1%	6.4%	8.3%	10.0%	13.1%	8.1%
Fluoroquinolones group 2 (levo- and moxifloxacin)	0.0%	0.0%	0.0%	0.1%	0.6%	1.5%	2.0%	2.7%	3.4%	4.3%	5.2%	3.2%
Fluoroquinolones group 3 (e.g., ofloxacin)	0.0%	0.0%	0.0%	0.0%	0.3%	0.8%	0.7%	0.7%	0.9%	1.0%	1.5%	0.9%
Metronidazole	0.0%	0.0%	0.1%	0.3%	0.4%	0.9%	1.5%	1.7%	2.3%	2.4%	1.9%	1.6%
Nitrofurantoin / fosfomycin / nitroxoline	0.2%	0.2%	0.7%	1.4%	5.6%	9.1%	8.4%	6.5%	6.5%	6.7%	10.2%	7.2%
Sulfonamides / trimethoprim	4.0%	3.1%	4.3%	4.1%	4.1%	3.9%	3.2%	3.1%	4.0%	4.7%	7.4%	4.8%
Tetracyclines	0.0%	0.0%	0.1%	2.1%	7.1%	7.8%	7.5%	7.0%	7.3%	7.0%	5.5%	6.0%
Other	0.1%	0.1%	0.1%	0.3%	0.3%	0.2%	0.2%	0.2%	0.1%	0.2%	0.3%	0.2%

[a] Prescriptions per 1000 persons/year; age- and sex-standardized using the German population on December 31, 2017 as reference.

Table S3b: Proportional distribution of antibiotic prescriptions by antibiotic subgroup in 2018 among females.

	Age group in years											Overall
	**0–1**	**2–5**	**6–9**	**10–14**	**15–17**	**18–24**	**25–34**	**35–44**	**45–54**	**55–64**	**≥65**	
Standardized prescription rate[a]	*282.7*	*558.3*	*383.1*	*253.9*	*437.0*	*560.8*	*504.0*	*526.8*	*488.4*	*533.7*	*576.2*	*511.0*
Combinations of aminopenicillins	4.2%	3.8%	3.8%	4.5%	4.8%	4.6%	5.0%	5.6%	6.0%	6.6%	7.5%	6.0%
Other combinations of aminopenicillins and antistaphylococcal penicillins	0.0%	0.0%	0.0%	0.2%	0.1%	0.1%	0.4%	0.3%	0.2%	0.1%	0.1%	0.2%
Amoxicillin	37.5%	31.4%	22.7%	21.5%	18.8%	15.5%	16.2%	16.4%	14.5%	13.9%	10.7%	15.2%
Phenoxymethylpenicillin	3.8%	10.8%	15.7%	12.4%	9.1%	7.1%	6.1%	4.9%	2.9%	2.5%	1.9%	4.5%
Other basic penicillins	1.9%	5.2%	6.5%	2.6%	0.3%	0.5%	0.5%	0.4%	0.5%	0.5%	0.8%	1.0%
Cephalosporins, first generation	0.5%	1.3%	1.9%	1.3%	0.6%	0.3%	0.4%	0.3%	0.3%	0.3%	0.2%	0.4%
Cephalosporins, second generation	31.9%	27.6%	25.3%	24.3%	17.0%	13.5%	14.4%	14.7%	13.1%	12.6%	12.5%	14.6%
Cephalosporins, third generation	8.5%	5.7%	4.8%	4.0%	2.9%	2.1%	2.0%	2.1%	2.1%	2.2%	2.6%	2.6%
Cephalosporins, fourth generation	0.0%	0.0%	0.0%	0.0%	0.0%	0.0%	0.0%	0.0%	0.0%	0.0%	0.0%	0.0%
Macrolides / lincosamides	6.5%	9.0%	10.9%	17.3%	20.2%	19.5%	20.3%	22.6%	23.1%	21.5%	14.6%	18.7%
Fluoroquinolones group 1 (ciprofloxacin)	0.2%	0.1%	0.3%	1.0%	3.7%	6.9%	6.5%	6.6%	8.4%	9.5%	12.3%	8.2%
Fluoroquinolones group 2 (levo- and moxifloxacin)	0.0%	0.0%	0.0%	0.1%	0.6%	1.5%	1.8%	2.4%	3.1%	3.8%	4.5%	2.9%
Fluoroquinolones group 3 (e.g., ofloxacin)	0.0%	0.0%	0.0%	0.0%	0.5%	1.2%	1.0%	1.0%	1.2%	1.3%	1.9%	1.2%
Metronidazole	0.0%	0.0%	0.1%	0.2%	0.5%	1.0%	1.5%	1.7%	2.1%	2.3%	1.9%	1.6%
Nitrofurantoin / fosfomycin / nitroxoline	0.2%	0.4%	1.2%	2.5%	9.3%	14.2%	13.3%	10.6%	10.8%	10.8%	14.9%	11.5%
Sulfonamides / trimethoprim	4.7%	4.6%	6.4%	5.7%	5.7%	5.2%	4.2%	4.0%	5.0%	5.6%	8.6%	5.9%
Tetracyclines	0.0%	0.0%	0.1%	2.2%	5.7%	6.6%	6.2%	6.2%	6.7%	6.3%	4.8%	5.4%
Other	0.1%	0.0%	0.1%	0.3%	0.2%	0.2%	0.1%	0.1%	0.1%	0.1%	0.2%	0.2%

[a] Prescriptions per 1000 persons/year; age-standardized using the German population on December 31, 2017 as reference.

Table S3c: Proportional distribution of antibiotic prescriptions by antibiotic subgroup in 2018 among males.

	Age group in years											Overall
	**0–1**	**2–5**	**6–9**	**10–14**	**15–17**	**18–24**	**25–34**	**35–44**	**45–54**	**55–64**	**≥65**	
Standardized prescription rate[a]	*343.3*	*577.8*	*350.5*	*219.8*	*284.3*	*292.6*	*286.5*	*357.9*	*356.7*	*402.8*	*478.7*	*371.3*
Combinations of aminopenicillins	4.8%	4.0%	4.4%	4.8%	5.9%	7.2%	7.8%	8.3%	9.0%	9.9%	11.5%	8.7%
Other combinations of aminopenicillins and antistaphylococcal penicillins	0.0%	0.0%	0.0%	0.0%	0.1%	0.1%	0.1%	0.1%	0.1%	0.1%	0.2%	0.1%
Amoxicillin	39.7%	32.7%	25.6%	23.7%	22.7%	21.4%	20.6%	20.4%	19.1%	17.3%	13.6%	19.5%
Phenoxymethylpenicillin	3.7%	11.8%	16.5%	12.8%	9.9%	9.1%	7.4%	6.1%	3.9%	3.1%	2.4%	5.7%
Other basic penicillins	1.9%	5.7%	6.6%	2.5%	0.2%	0.1%	0.3%	0.3%	0.3%	0.3%	0.4%	0.9%
Cephalosporins, first generation	0.5%	1.4%	2.2%	1.5%	0.6%	0.4%	0.4%	0.3%	0.3%	0.3%	0.3%	0.5%
Cephalosporins, second generation	31.6%	28.0%	25.7%	25.4%	18.4%	15.4%	14.5%	14.8%	14.0%	13.4%	13.8%	16.1%
Cephalosporins, third generation	6.8%	5.0%	3.8%	3.6%	2.7%	2.3%	2.3%	2.3%	2.2%	2.3%	2.7%	2.7%
Cephalosporins, fourth generation	0.0%	0.0%	0.0%	0.0%	0.0%	0.0%	0.0%	0.0%	0.0%	0.0%	0.0%	0.0%
Macrolides / lincosamides	7.1%	9.5%	12.4%	19.7%	24.9%	25.3%	25.3%	25.6%	24.6%	21.9%	16.0%	20.6%
Fluoroquinolones group 1 (ciprofloxacin)	0.1%	0.1%	0.3%	0.7%	2.0%	4.0%	5.5%	6.1%	8.2%	10.6%	14.2%	8.0%
Fluoroquinolones group 2 (levo- and moxifloxacin)	0.0%	0.0%	0.0%	0.1%	0.5%	1.6%	2.3%	3.1%	3.8%	4.9%	6.3%	3.6%
Fluoroquinolones group 3 (e.g., ofloxacin)	0.0%	0.0%	0.0%	0.0%	0.1%	0.2%	0.3%	0.3%	0.4%	0.6%	0.9%	0.5%
Metronidazole	0.0%	0.0%	0.1%	0.3%	0.3%	0.7%	1.3%	1.8%	2.4%	2.5%	1.9%	1.6%
Nitrofurantoin / fosfomycin / nitroxoline	0.2%	0.1%	0.2%	0.3%	0.4%	0.4%	0.4%	0.5%	0.8%	1.2%	3.0%	1.2%
Sulfonamides / trimethoprim	3.5%	1.7%	2.1%	2.5%	1.9%	1.5%	1.7%	1.9%	2.5%	3.6%	5.7%	3.2%
Tetracyclines	0.0%	0.0%	0.1%	2.0%	9.1%	9.9%	9.5%	8.0%	8.1%	7.9%	6.7%	6.9%
Other	0.1%	0.1%	0.2%	0.2%	0.3%	0.3%	0.2%	0.2%	0.2%	0.2%	0.4%	0.3%

[a] Prescriptions per 1000 persons/year; age-standardized using the German population on December 31, 2017 as reference.

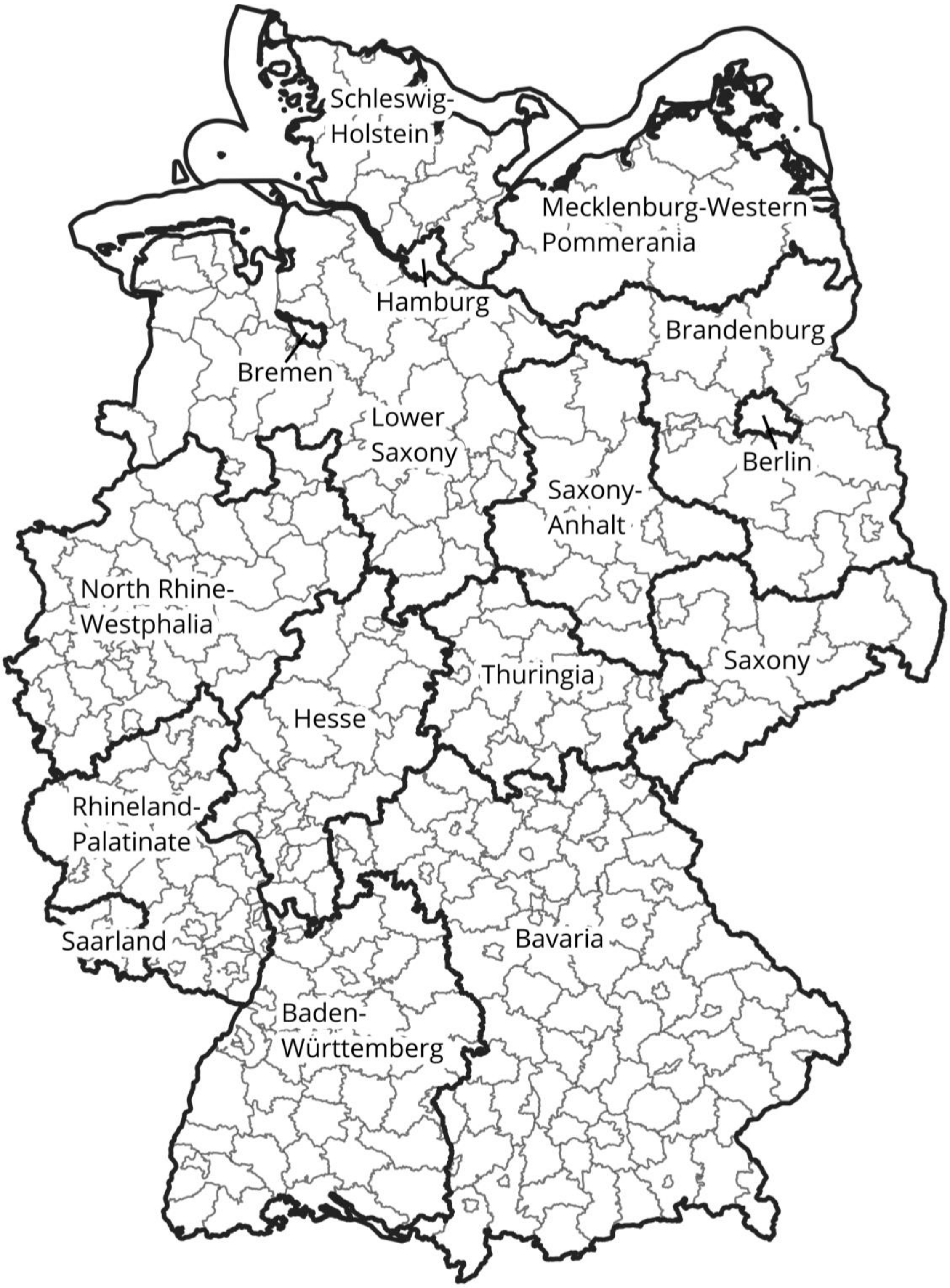

Source: © GeoBasis-DE / BKG 2017

Figure S1: Map of Germany with the 16 federal states.

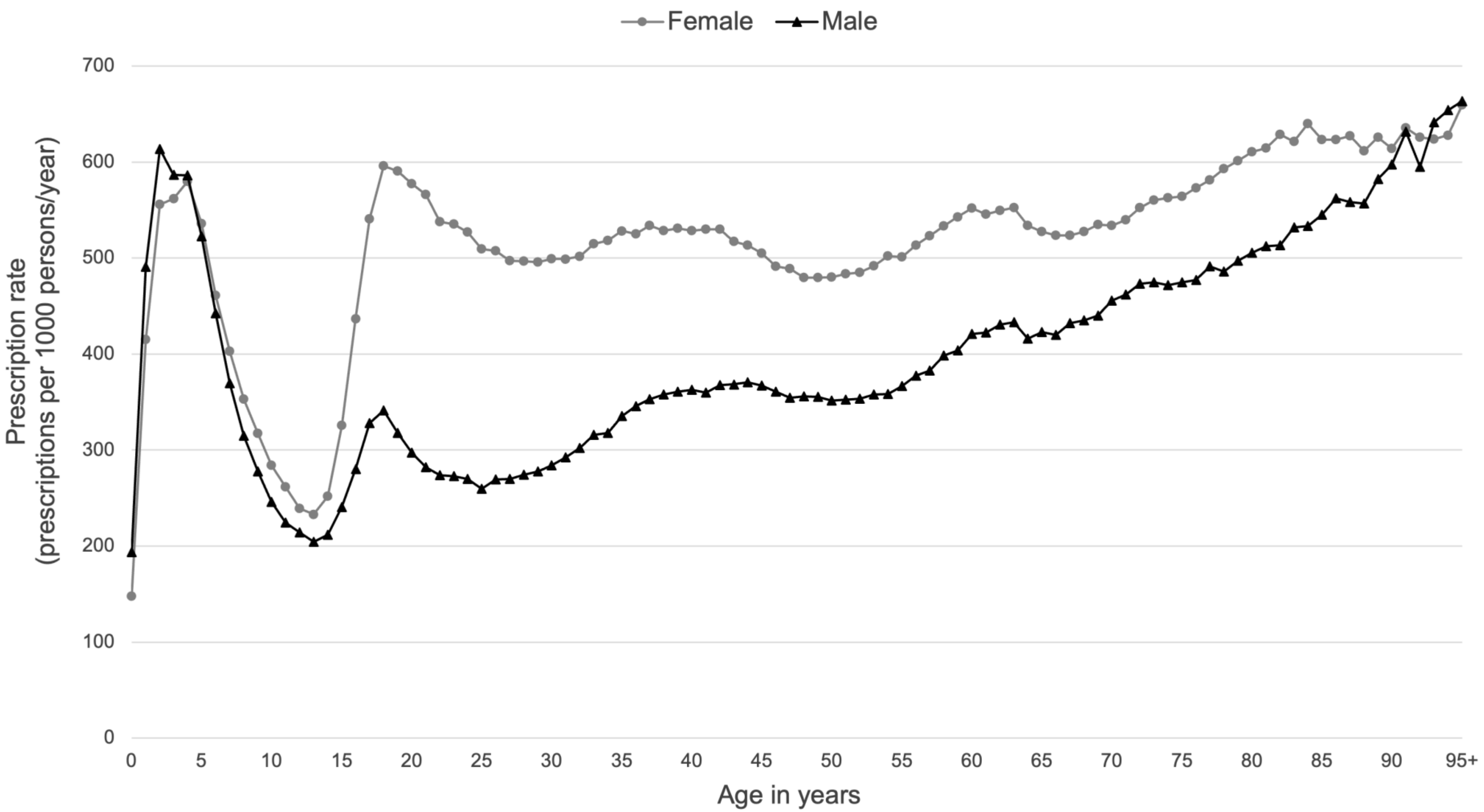

Figure S2: Age-specific prescription rates (prescriptions per 1000 persons/year) of antibiotics by sex in 2018.

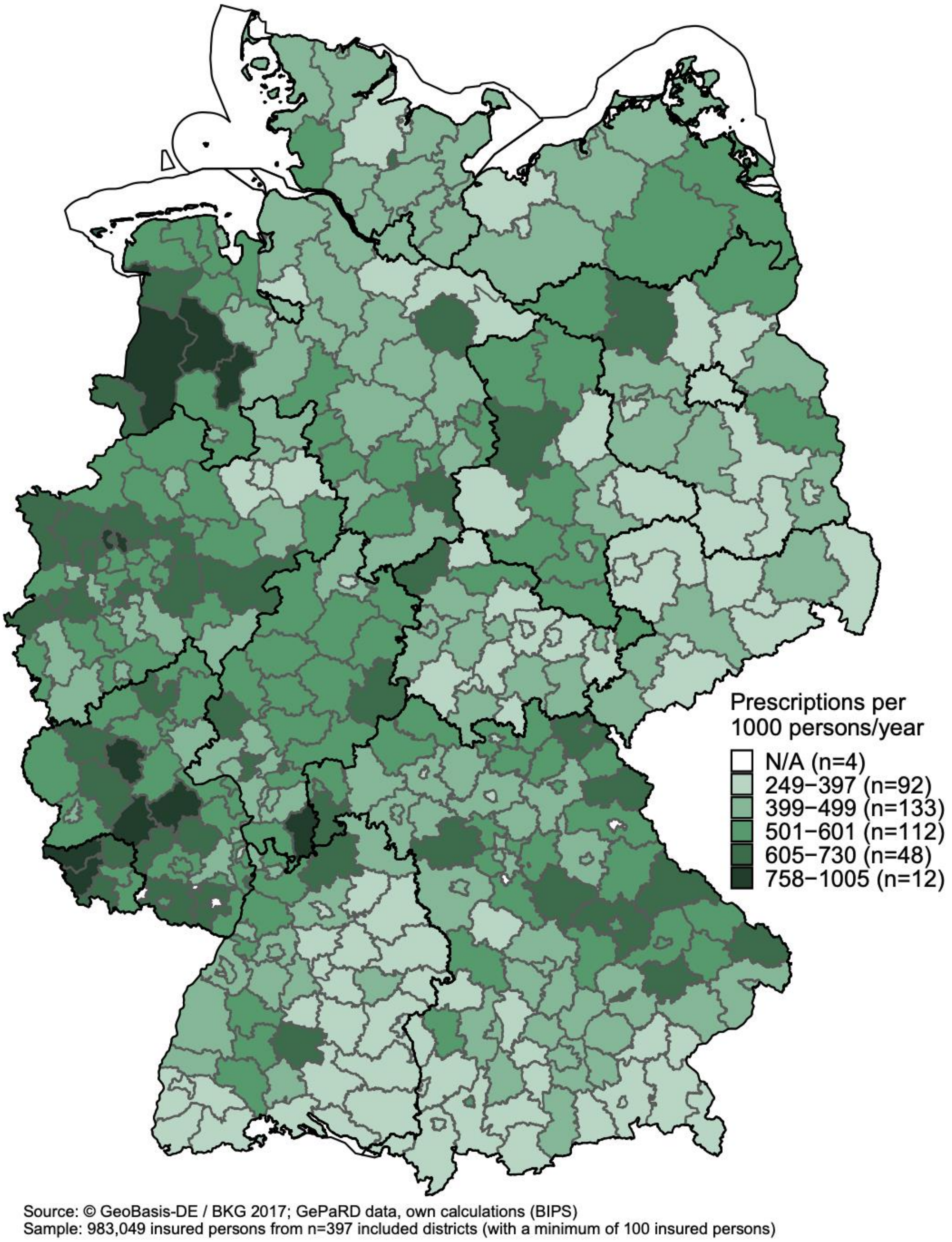

Figure S3: Age- and sex-standardized prescription rates (prescriptions per 1000 persons/year) of antibiotics among children aged ≤6 years by district in 2018.

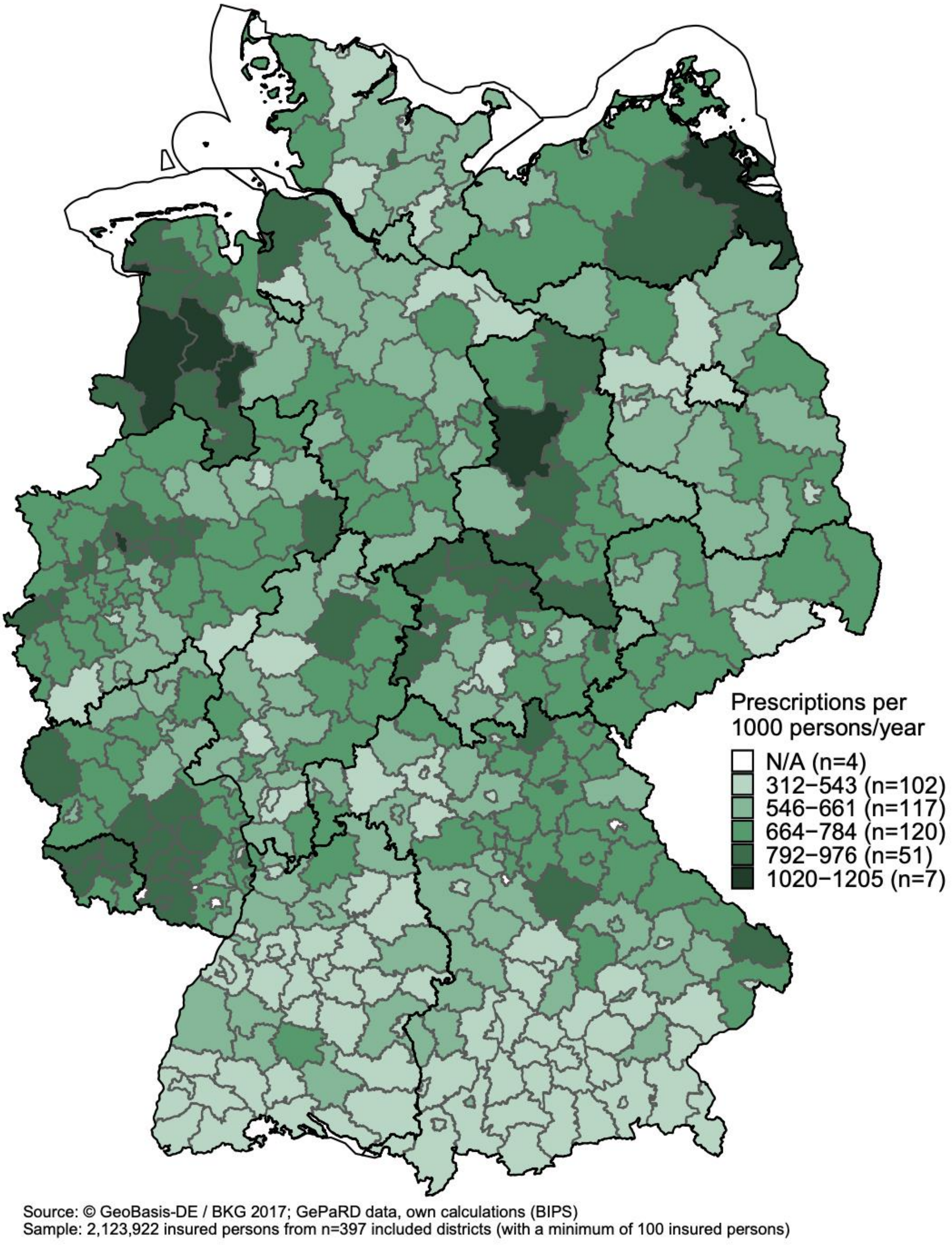

Figure S4: Age- and sex-standardized prescription rates (prescriptions per 1000 persons/year) of antibiotics among children and adolescents aged 0–17 years by district in 2010.

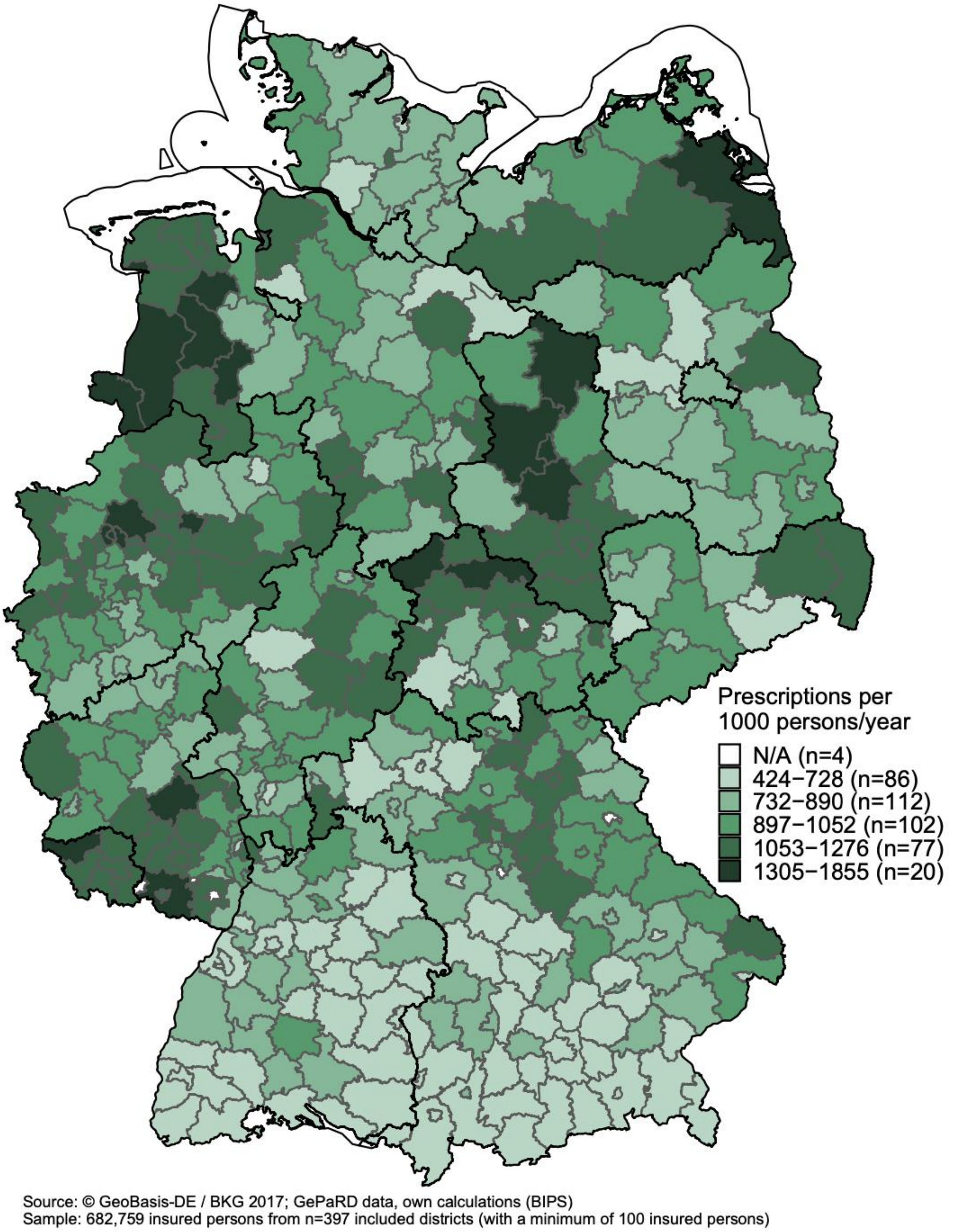

Figure S5: Age- and sex-standardized prescription rates (prescriptions per 1000 persons/year) of antibiotics among children aged ≤6 years by district in 2010.

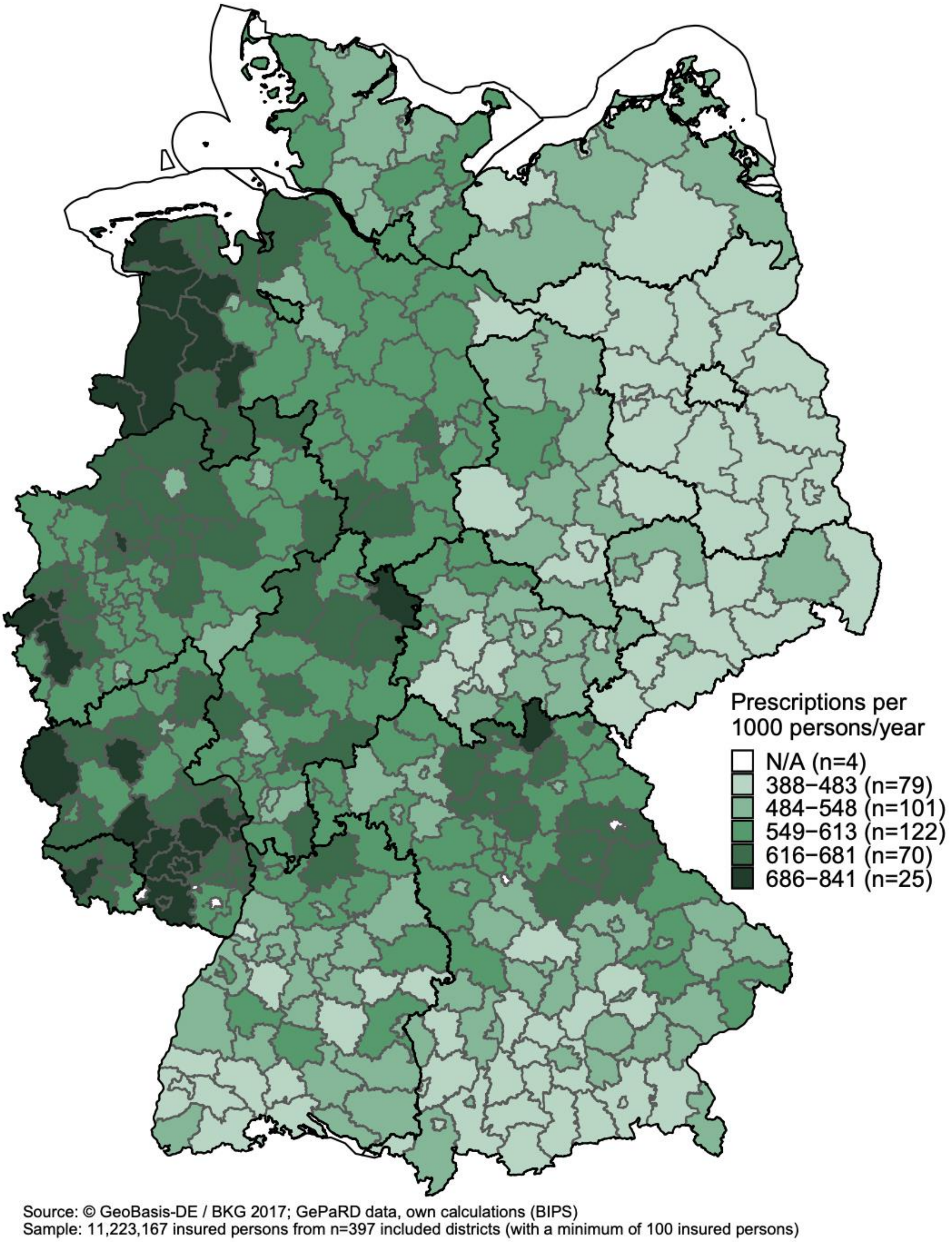

Figure S6: Age- and sex-standardized prescription rates (prescriptions per 1000 persons/year) of antibiotics among adults aged ≥18 years by district in 2010.

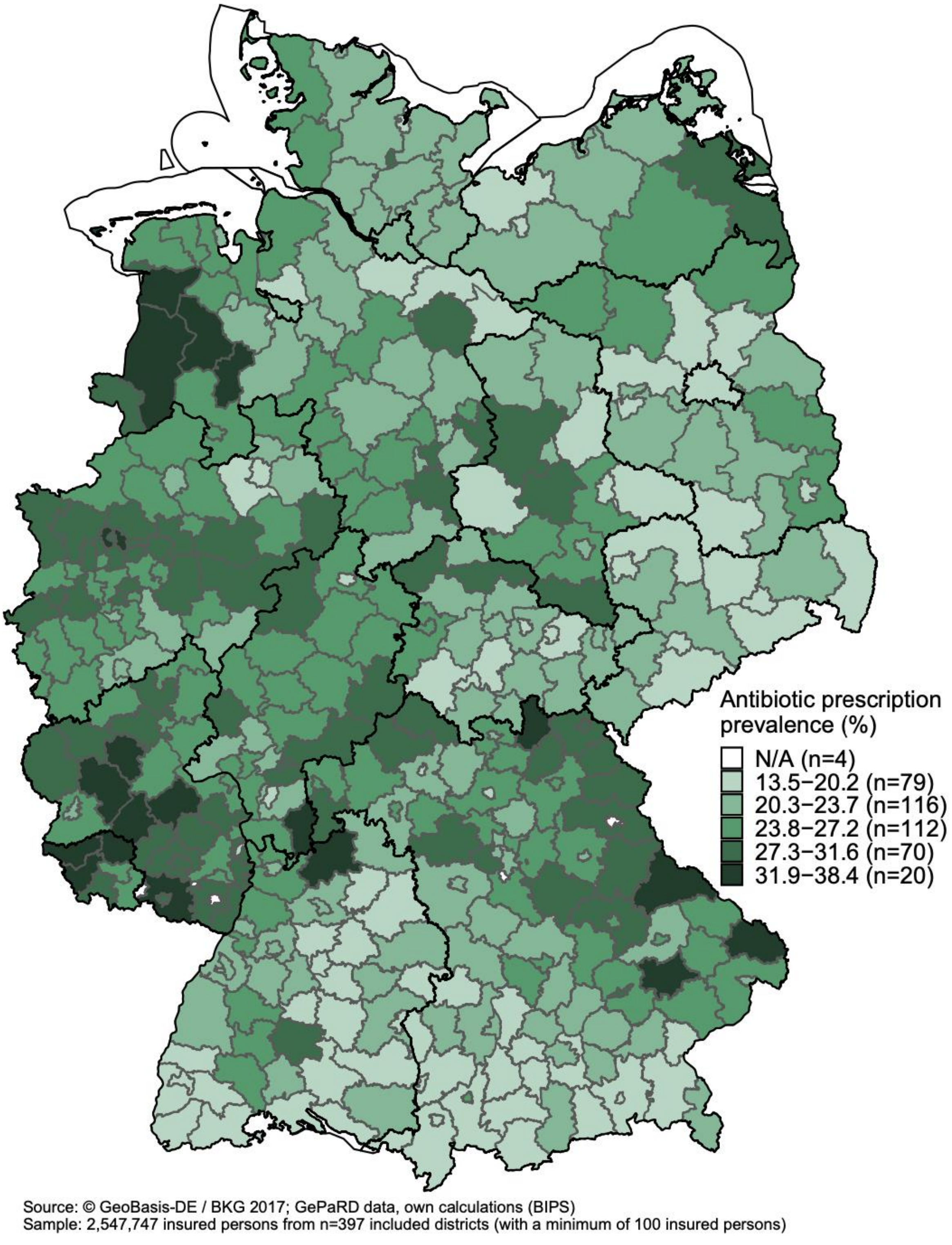

Figure S7: Age- and sex-standardized prescription prevalence (%) of antibiotics among children and adolescents aged 0–17 years by district in 2018 (see **Text S1** below for a description).

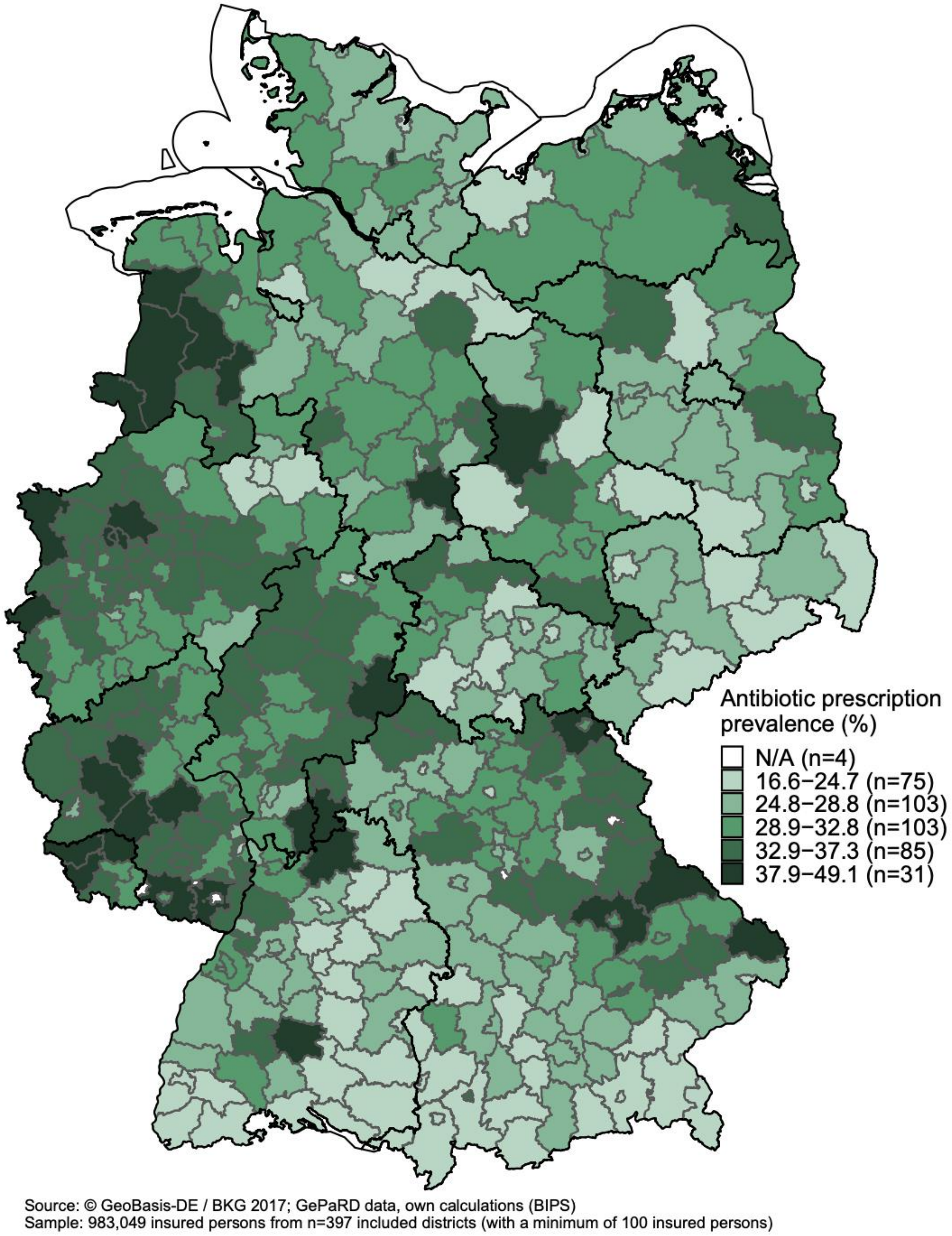

Figure S8: Age- and sex-standardized prescription prevalence (%) of antibiotics among children aged ≤6 years by district in 2018 (see **Text S1** below for a description).

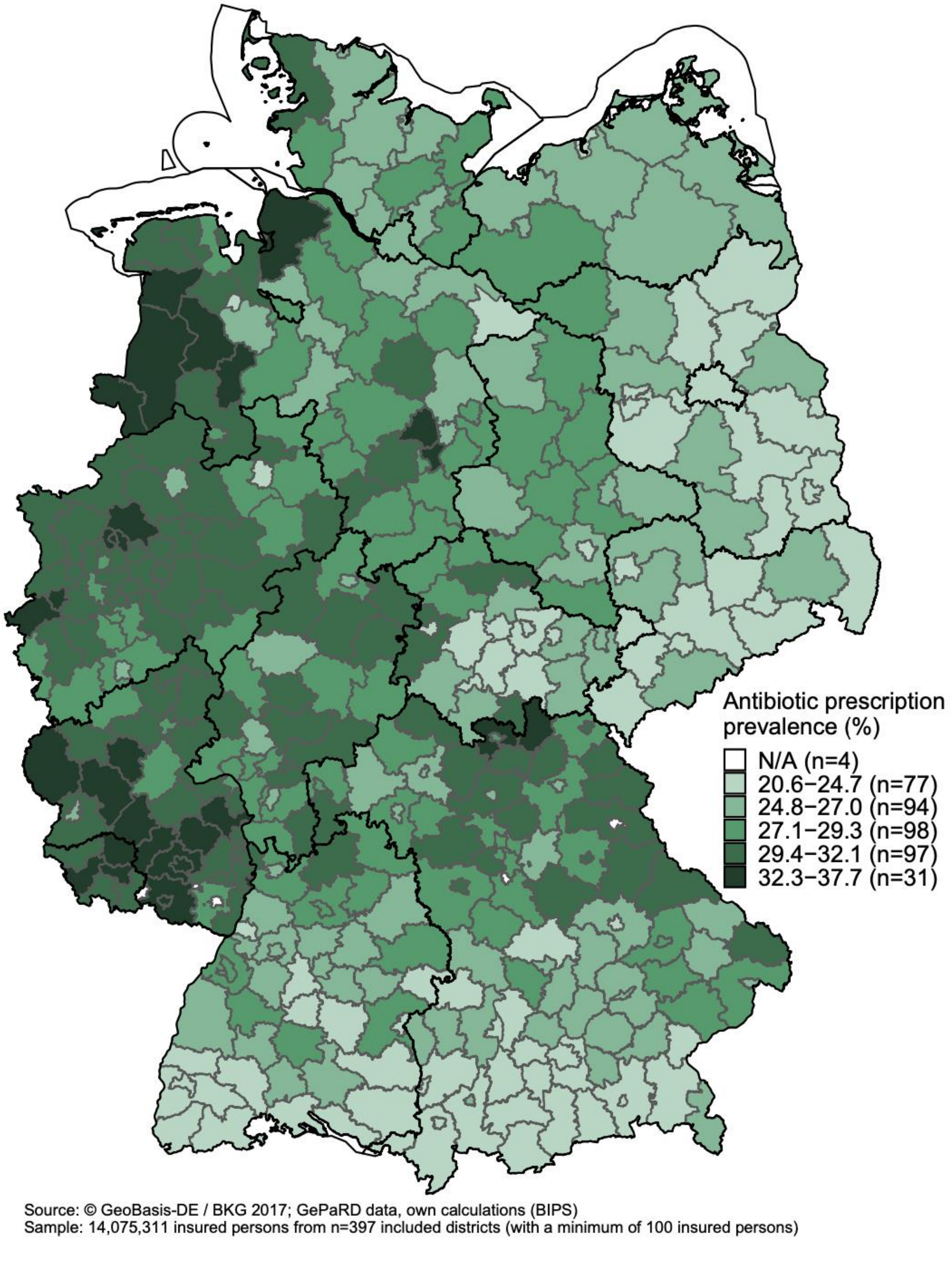

Figure S9: Age- and sex-standardized prescription prevalence (%) of antibiotics among adults aged ≥18 years by district in 2018 (see **Text S1** below for a description).

Text S1: Regional variations in antibiotic prescribing based on the prescription *prevalence* in 2018 (referring to Figures S7–S9).

When comparing the regional variation based on the prescription rate with that based on the prescription prevalence, the geographical pattern was similar for all investigated age groups, i.e., 0–17 years (**Figure S7**), ≤6 years (**Figure S8**), ≥18 years (**Figure S9**). However, in all age groups, the range of the values differed based on the prescription prevalence as compared with the prescription rate. The largest variation was observed in the age group comprising children ≤6 years: The prescription prevalence ranged from 16.6 to 49.1% (**Figure S7**), i.e., it differed by a factor of 3—as compared to a factor of >4 based on the prescription rate (**Figure S3**).
